# 3500 years of shellfish mariculture on the Northwest Coast of North America

**DOI:** 10.1371/journal.pone.0211194

**Published:** 2019-02-27

**Authors:** Nicole F. Smith, Dana Lepofsky, Ginevra Toniello, Keith Holmes, Louie Wilson, Christina M. Neudorf, Christine Roberts

**Affiliations:** 1 Hakai Institute, Heriot Bay, BC, Canada; 2 Independent Archaeologist, Victoria, BC, Canada; 3 Department of Archaeology, Simon Fraser University, Burnaby, BC, Canada; 4 We Wai Kai Nation, Quathiaski Cove, BC, Canada; 5 Department of Geography and the Environment, University of the Fraser Valley, Abbotsford, BC, Canada; 6 Wei Wai Kum First Nation, Campbell River, BC, Canada; Max Planck Institute for the Science of Human History, GERMANY

## Abstract

Ancient systems of mariculture were foundations of social-ecological systems of many coastal Indigenous Peoples. However, since such systems either do not leave tangible remains in the archaeological record, and/or are hard to date, we know little about their development and use. Clam gardens, traditional mariculture features located within the intertidal zone along the Northwest Coast of North America, are composed of a rock wall positioned at the low tide mark and a flattened terrace on the landward side of the wall. Because these features are largely composed of rock and sediment, and have complex formation histories, they can be difficult to age. On northern Quadra Island, British Columbia, we identify three variations in clam garden form, constructed in different geomorphological settings, each of which require different sampling approaches to obtain ages on construction and ongoing use. To age the clam gardens, we consider radiocarbon dating of invertebrates that inhabit beach deposits (both pre- and post-garden construction), and the relationship of the gardens and clam samples to the local sea level history and taphonomic processes. Within our study area, we find clam gardens have been in use for 3500 years, likely corresponding to other social and ecological changes of the time. These data allow us to formulate guidelines on samples most suitable to constrain the age of initial and on-going wall construction and use of clam gardens, which can be extrapolated to dating other ancient mariculture features in other regions. Such dating programs are the foundation for understanding the long-term development of traditional marine management practices and how they are situated in broader social-ecological systems.

## 1.0 Introduction

Indigenous and local peoples around the world have developed a suite of management techniques to maintain and enhance culturally important resources [1–3]. However, documenting such techniques in the archaeological and paleoecological records, and thus the larger social-ecological contexts in which they are embedded, can be elusive [4, 5]. Clam gardens, an ancient mariculture technique used by Indigenous People of the Northwest Coast of North America, provide a unique opportunity to study the tangible aspects of ancient marine management systems. These rock-walled intertidal terraces, in combination with a variety of cultivation techniques, enhanced clam productivity and abundance through a variety of mechanisms [6–9]. In the past, as today, these features were linked to the governance, livelihoods, and identity of coastal First Nations from Alaska to Washington State [6, 8, 10, 11]. The persistence of these rock features in the archaeological record of the region allows us to track the development of this form of management through time and space.

As inorganic (rock) features situated in a largely organic and continually regenerative context (the intertidal ecosystem), dating clam gardens presents both unique opportunities as well as challenges [8]. Although datable organisms are abundant in intertidal ecosystems, clam garden researchers are faced with the archaeological challenge of temporally linking organic remains with the rock wall construction event. Making this link is complicated by the constant and sometimes dramatic wave action associated with intertidal systems, the ability of many marine organisms to burrow, the persistence of millennia-old shells in beach deposits, the degradation of datable material, increased sedimentation from logging, and the ongoing harvesting of clams and wall maintenance by diggers over generations.

Here we build on our previous research on the dating of clam garden construction and use [8, 12] and thus the role of clam gardens in ancient social-ecological systems. Our expanded survey and excavations demonstrate that past clam gardeners built three different types of clam gardens, depending on the pre-existing beach geology and ecology. While we categorize clam gardens into three discrete forms for ease of sampling, the greater importance of noting these forms is that they reflect the breadth and ingenuity of clam garden engineering.

**Fig 1:**
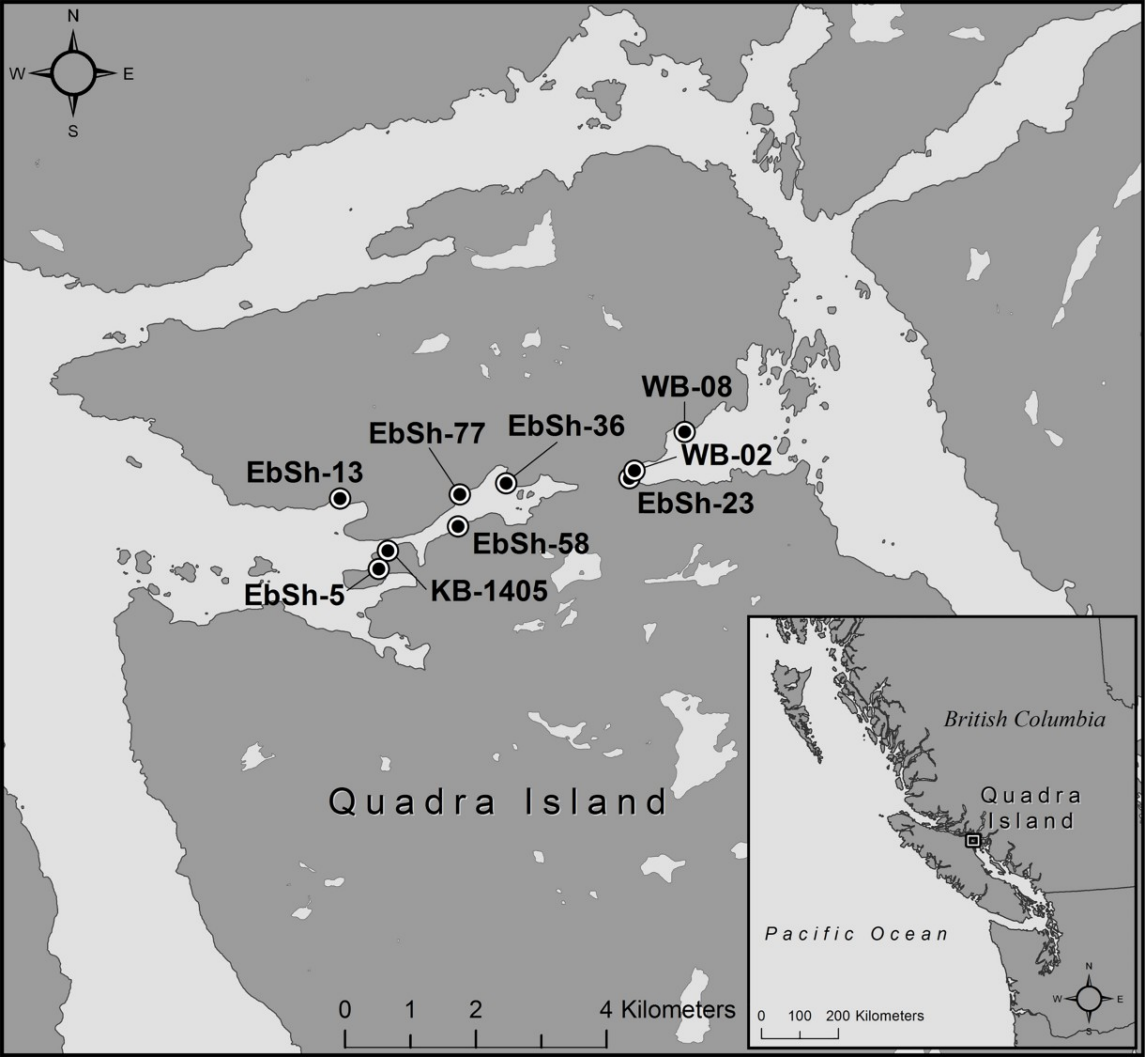
The study area on northern Quadra Island, British Columbia, showing the nine sites from where radiocarbon dates were extracted. Imagery contains information licensed under the Open Government License-Canada and from Natural Earth. Map data also sourced from OpenStreetMap contributors.

Based on radiocarbon dating from nine clam gardens on Northern Quadra Island, British Columbia (Fig 1), as well as our understanding of the local sea level history and taphonomic processes, we assess different methods and sample types for dating clam gardens in a variety of settings and determine that some clam gardens in our study area are over 3500 years old. By providing accurate ages for clam gardens, our analyses move us closer to understanding the social and ecological context of clam gardens during the latter half of the Holocene, and provide insights into dating other mariculture features elsewhere in the world.

## 2.0 Study area and methods

Quadra Island is located off the northeast coast of Vancouver Island in southwest British Columbia, Canada in the traditional territories of the Laich-Kwil-Tach and northern Coast Salish peoples. Our study area at the north end of Quadra Island (Fig 1) focuses on Kanish and Waiatt Bays, two large water bodies separated by a narrow 750 m stretch of land traversed easily by foot. Recent refinement of the relative sea level (RSL) history [13, 14] indicates that sea level at the north end of Quadra Island fell rapidly following the last glacial maximum from a height of over 195 m to within approximately 2 to 4 m of modern levels by 12,300 years ago [13]. This drop was followed by a minor transgression before RSL fell gradually from about 3 m above modern around 10,500 years ago, down to about 1 m above modern by 4000 years. By 2000 years ago, sea level was within 35 cm of modern day levels and then gradually declined to current levels [13, 14]. Tracking the progression of RSL change is vital for locating shoreline proximal sites and for understanding clam garden development through time.

Not surprisingly, human habitation of the study area is closely tied to the local sea level history. Lithic sites on raised beach terraces in the study area date back to 12,900 years ago based on dated deltaic deposits and relative sea level position [13]. Due in part to dissolution of shell from acidic ground water, dense shell middens do not appear in the archaeological record until around 5,000 years ago. After that time, large settlement sites become more apparent in the later Holocene (Lepofsky unpublished data).

With over 15 km of rock walls [15] and more than 15 ha of clam habitat [9], Kanish and Waiatt bays house among the highest density of clam gardens currently known on the Northwest Coast. These mariculture features are found both on beaches with soft sediments containing pre-existing clam habitat, as well as along bedrock shorelines where boulder wall constructions have encouraged new beaches and clam habitat to form. Gardens tend to be located along the sides of semi-protected inlets with strong tidal currents and away from the heads of bays or deltas. Local knowledge indicates that First Nations maintained clam garden walls into the 20^th^ century. In particular, Keekus (Elizabeth Harry) told historian Judith Williams in 1993 that Waiatt Bay had the “best butter clams”, and that she replaced rocks that had fallen from the terrace walls when she was digging there [16, 17]. Today, local First Nations’ community members dig clams from many of these beaches, as do visiting tourists and non-Indigenous residents.

### 2.1 Characteristics of clam gardens

Understanding the general characteristics and formation history of clam gardens is a requisite step for dating wall construction and maintenance events and thus for understanding the local development of mariculture. In general, a clam garden consists of a boulder wall constructed at or near the lowest tide line. Wall height was augmented and maintained through on-going rolling of rocks down to the lowest intertidal whenever people dug clams [6]. The wall increases butter clam (*Saxidomus gigantea*) and littleneck (*Protothaca staminea)* habitat by creating a shell-hash and sediment filled terrace at particular tidal zones [8, 18]. Today, these species of clams can be 2–4 times more productive in clam garden beaches than in non-walled beaches in the same area [7, 19].

**Fig 2:**
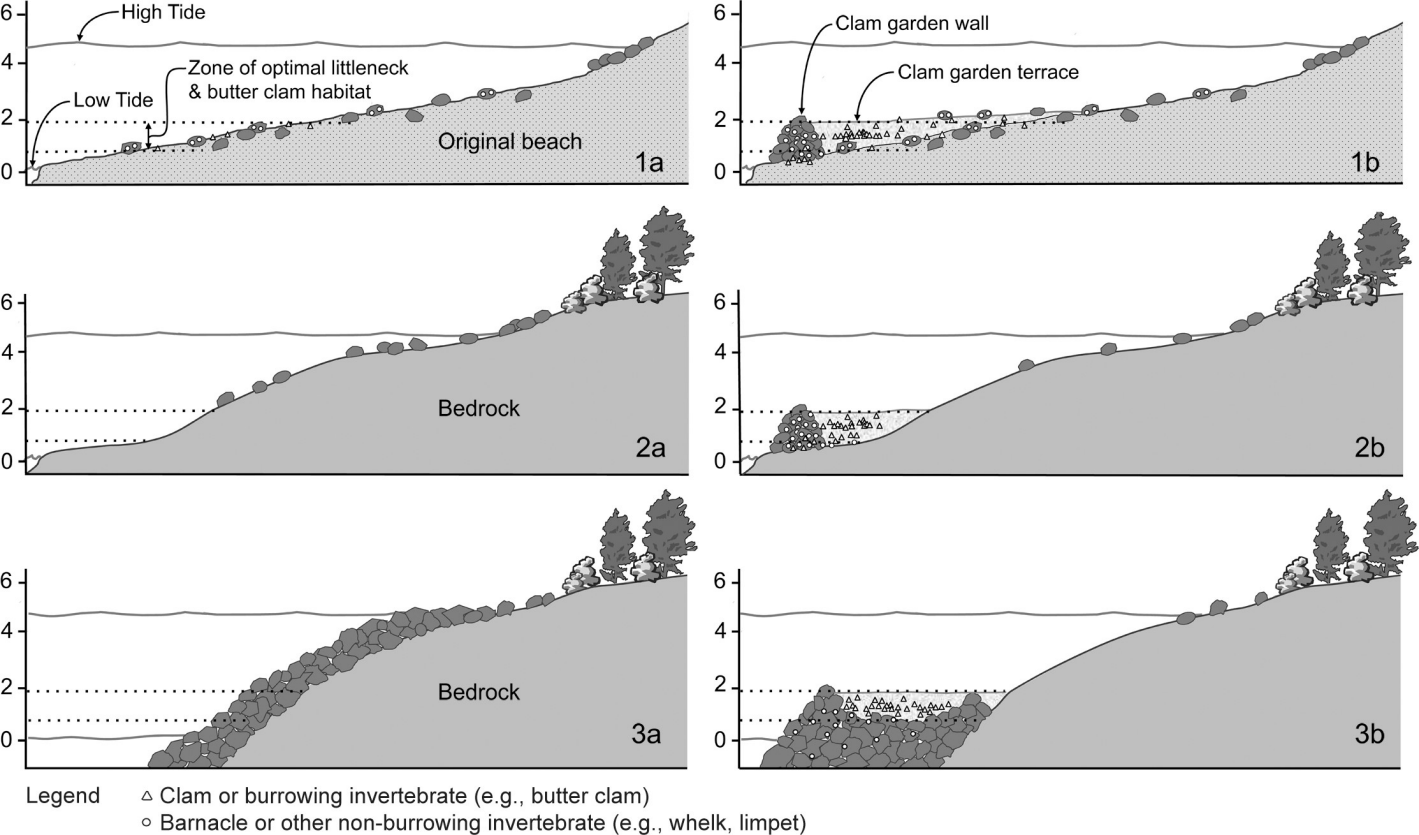
Sequence of construction of three different clam garden forms, based on northern Quadra Island sites. Falling sea level during the Holocene means that the base of many clam garden walls, particularly those built on soft sediment, are often exposed during the lowest tidal windows today. Form 1. Clam gardens built on soft sediment beaches; (1a) Original unmodified beach surface with existing clam habitat prior to wall construction; (1b) Clam garden wall and terrace on same beach. Note clams and barnacle scars from original beach covered by clam garden wall and terrace sediment. Note also some boulders with barnacle scars from original beach moved into clam garden wall during construction. Stippled light coloured sediment behind wall indicates shell hash and coarse sediment that accumulated after wall was built, thereby expanding clam habitat; Form 2. Clam gardens built on bedrock shelf; (2a) Original bedrock shoreline; (2b) Wall built on same bedrock to create clam garden terrace. Note base of wall is not always accessible at low tide; Form 3. Clam gardens built on steep eroding bedrock shoreline; (3a) Original steep, eroding bedrock shoreline. (3b) Clam garden wall and terrace in same location. Rocks from mid to upper intertidal and supratidal are moved down slope to create levelled terrace at the elevation for optimal clam habitat. Note base of wall is usually not accessible at low tide.

**Fig 3:**
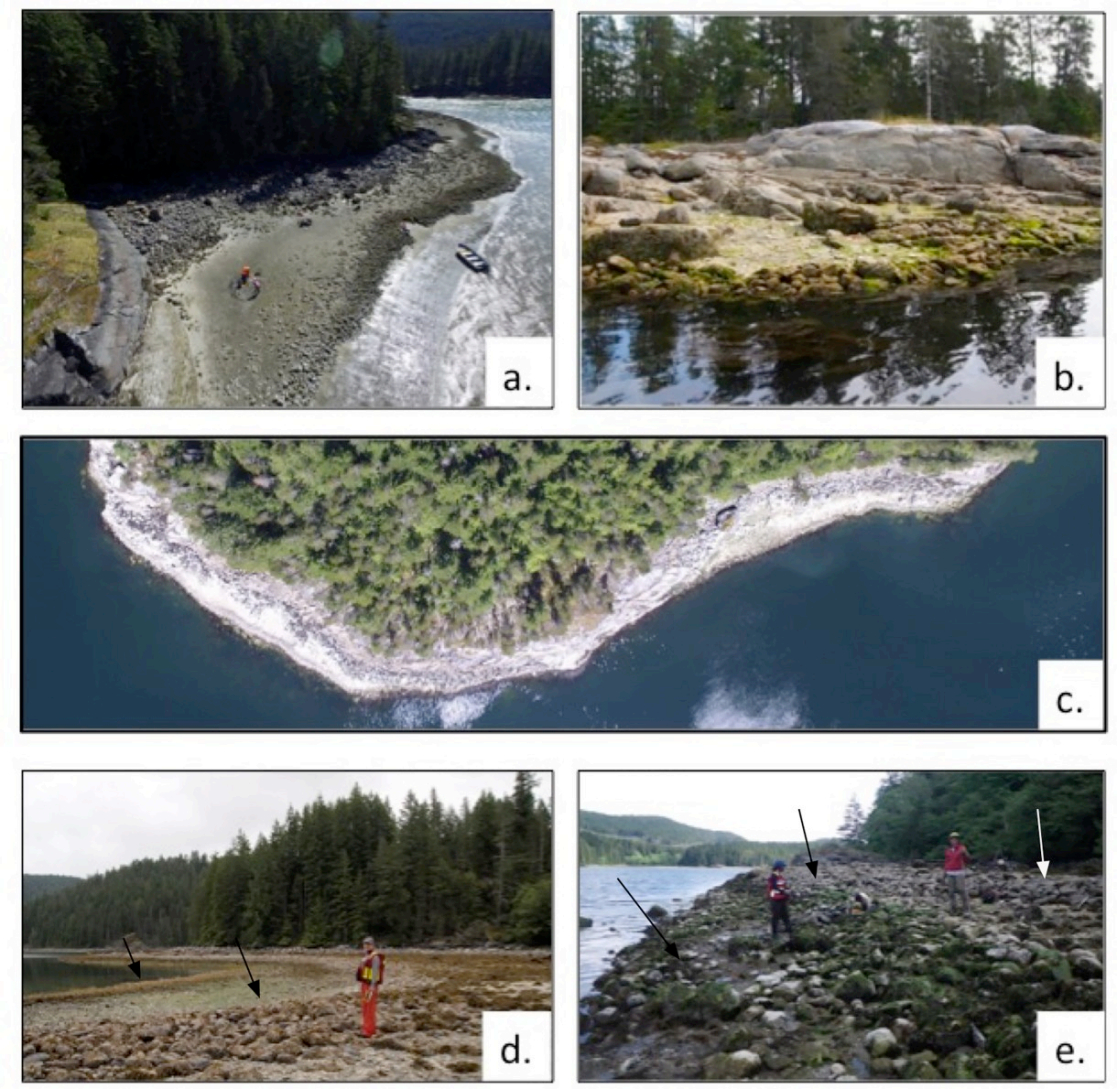
Different clam garden forms and select study sites on Quadra Island. a) EbSh-36, Form 1 clam garden built on soft sediment beach, b) Form 2 clam garden built on bedrock, c) WB08, Form 3 clam garden created by levelling boulders along steep, eroding bedrock shoreline, d) EbSh-5, Form 1, showing two walls at different tidal heights, e) EbSh-58, Form 1, showing three walls at different tidal heights.

Within the general form of clam gardens, we identify three variations in construction that occur in isolation and in various combinations, depending on the geomorphology of particular sites (Figs 2 and 3). The three forms are: Form 1- those built on soft sediment beaches with already existing clam habitat (Fig 2, Form 1 (a,b); Fig 3A); Form 2- those built on flat bedrock outcrops (Fig 2, Form 2 (a,b); Fig 3B); and Form 3- those created along a section of steep, eroding, bedrock shoreline where people have levelled the eroding boulders to create a flattened platform (Fig 2, Form 3 (a,b); Fig 3C). The majority of Form 1 gardens consist of a single wall at one tidal height, but some sites have multiple walls and terraces at different tidal heights (Fig 3, d and e). In Form 2 gardens, there may have been small pockets of sediment on the bedrock shelf that could have provided clam habitat prior to wall construction. In contrast, in Form 3, no prior clam habitat could have existed. As we discuss later, the creation of beach habitat where there was none prior is significant both in terms of cultural and natural history, and in the selection of suitable dating material. Collectively, these forms reflect that people engineered many possible geomorphic settings and ecosystems to maintain and increase the production of their staple marine foods.

### 2.2 Excavation and sample collection

We conducted subsurface testing, sample collection, and radiocarbon dating at nine clam garden sites, six in Kanish Bay and three in Waiatt Bay (Fig 1). Excavation took place during the lowest May tides (2013–2017) with tidal heights lower than +0.7 m. Archaeological work was conducted under permits #2010–0251 and #2014–0095 from the BC Archaeology Branch. All samples (if not consumed entirely by radiocarbon analysis) are presently housed at Simon Fraser University. No human subjects or vertebrates were involved in this study.

We focused part of our sampling effort on sites that we believed would yield dates associated with older clam gardens, when sea level was higher. That is, we looked for terraces whose current surfaces are above the productive butter clam zone (~+0.5 m; [20]), and/or for sites with multiple walls up the beach that might reflect the temporal sequence of clam garden building at a single location. In addition, we tried to sample beaches in different geomorphological contexts. Our initial sampling efforts focussed on Form 1 gardens but towards the end of our project we expanded our efforts to include Forms 2 and 3. While we tested fourteen gardens, we retrieved suitable dating samples from the nine gardens included in this paper.

**Fig 4:**
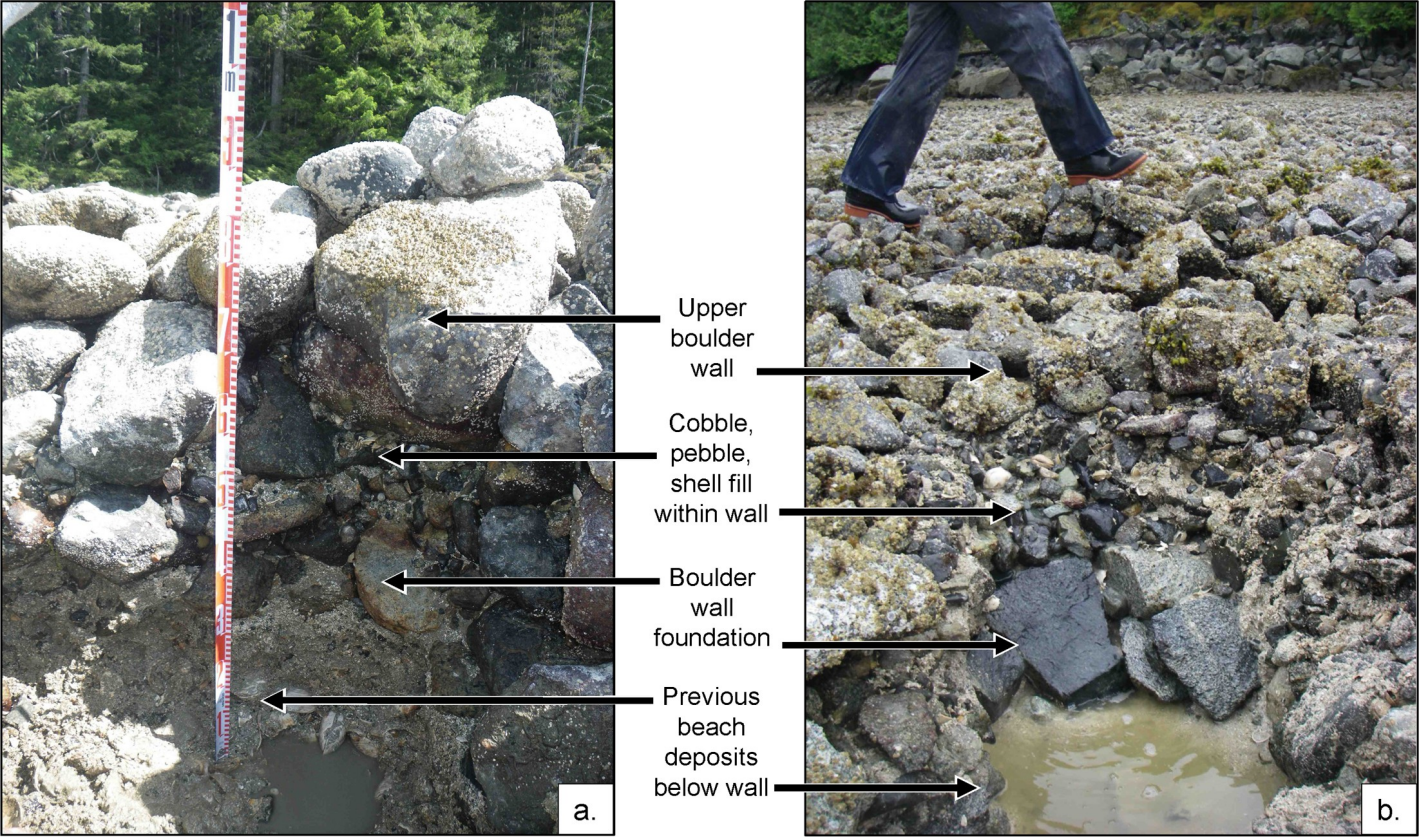
Clam garden wall stratigraphy as revealed in two wall trenches. (a) EbSh-36, Trench 1 (2014), and (b) EbSh-5 upper wall, Trench 2 (2013). Note the rounded and sub rounded boulders typically found in the intertidal zone (a), compared to the angular boulders in the wall foundation that are typical of eroding bedrock near the tree line (b).

Our strategy was to target dating contexts that yielded samples associated with the initial construction of the wall, and early use of the clam garden beach. To examine stratigraphy and extract datable samples at Form 1 clam gardens, we dug shovel tests in the terrace and excavated 1–1.5 m wide trenches within the wall (Fig 4). We sought datable material (such as barnacle scars, clams, whelks, limpets, and wood) at the base of the wall that could be temporally linked to initial wall construction, or for similar samples within the wall and terrace that could be associated with ongoing garden construction, maintenance, and use (i.e., adding rocks to the wall at each visit and the subsequent infilling of the terrace). When the water table allowed, we excavated through the wall or terrace and into underlying original beach deposits. On the original beach surface, we looked for organic materials that would have been trapped by wall construction or terrace infilling and therefore would provide a maximum age for when the clam garden wall was initiated.

In the Form 2 and 3 clam gardens where we could not access the base of the wall during low tide, we dug shovel tests in the terrace down to bedrock or boulder substrate. Within the terraces of all the Forms, our strategy was to find burrowing shellfish specimens that established themselves in the sediments that began accumulating after the initial wall was built. In Form 2 and 3 gardens, we also sought surface dwelling invertebrates that inhabited the bedrock or boulder base immediately prior to sediment infill. In Form 2 gardens built on bedrock shelves, we were cognizant that there could be naturally accumulated sediment and clams that could pre-date wall construction. In Form 3 walls, after some trial and error, we looked explicitly for portions of the terrace that were part of the newly constructed anthropogenic landform and could not have existed prior to the building of the wall–that is, those portions of the gardens that were built out beyond the original beach edge.

In all cases, we established datum markers within the excavation units that were tied to chart datum (Kanish Bay tidal range = 4.89 m, LLWLT = 0.01 m above chart datum; Waiatt Bay tidal range = 4.71 m, LLWLT = 0.06 m above chart datum). Depth below surface was recorded for each collected sample. Excavation locations were recorded with handheld GPS and mobile mapping units (horizontal accuracies of +/- 3 m and +/-2 m respectively), hand drawn site maps, or with survey grade GPS and drone technology (<10 cm accuracy).

### 2.3 Determining tidal heights

Our understanding of clam garden formation history, and our ability to examine the temporal relationship of dated samples to clam garden construction and past sea levels, required collecting precise elevations of the radiocarbon samples, wall bases, and wall heights. Elevation data were acquired using a survey grade GPS (Topcon GR5) and real time kinematic methods, with elevation values referenced to local chart datum tidal heights.

**Fig 5:**
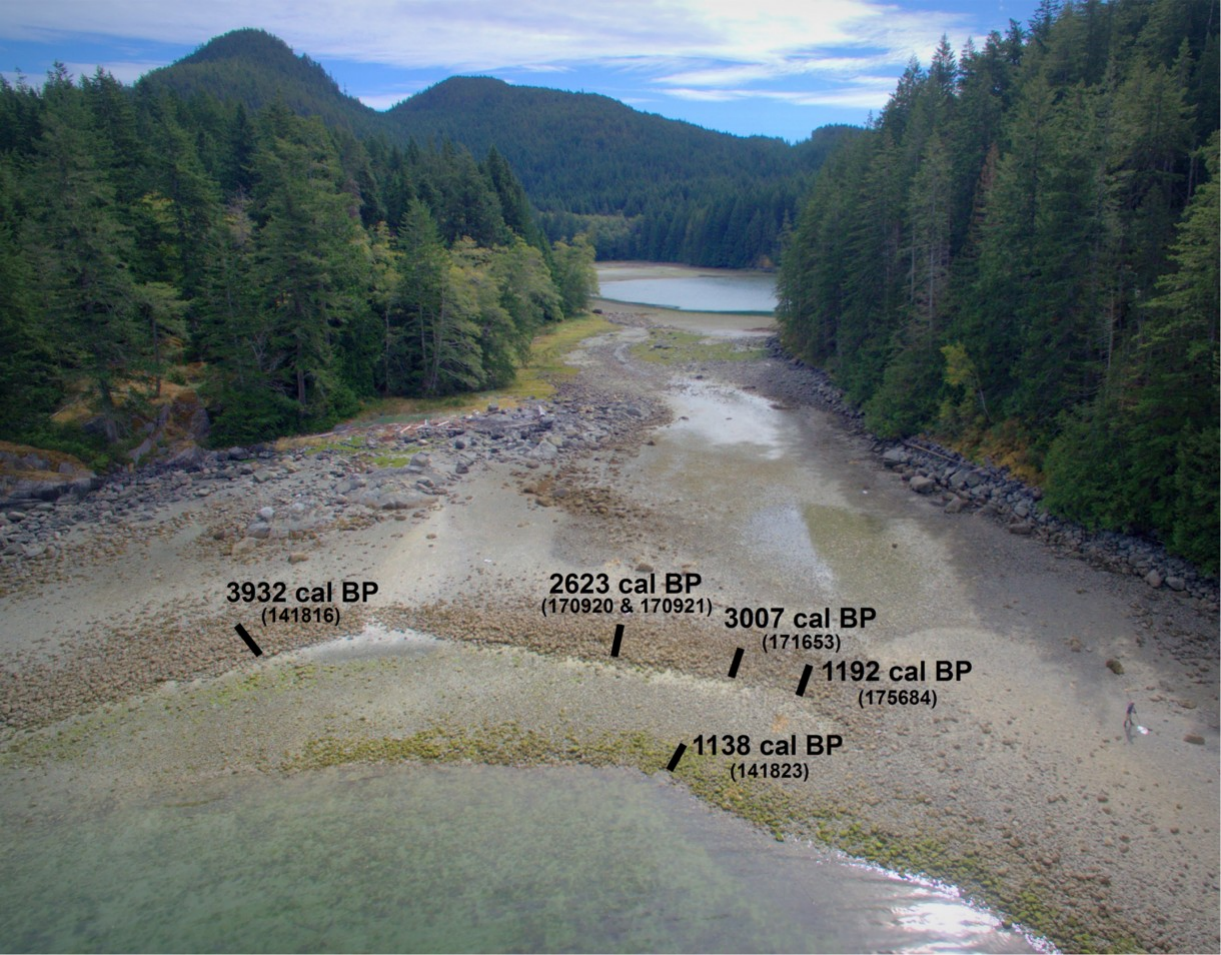
UAS imagery of site EbSh-5 showing excavation trench locations and radiocarbon dates from immediately below the wall.

A DJI Phantom 3 Professional Unoccupied Aerial System (UAS) was used to capture high-resolution imagery of the study area at low tide (<1.0 m), and positioned with high visibility ground control point (GCPs). Imagery and GCP data (Fig 5) were processed in structure-from-motion (SfM) software (Pix4D 4.0.25) to produce orthomosaic images, and digital surface models (DSM) of elevation. Elevations of all excavation units were extracted from the DSM.

Positional accuracy was calculated based on statistical analysis of our control and SfM software results. Taking into consideration compounding error from all survey and software sources a conservative vertical accuracy assessment was estimated to be < 10 cm for six of our sites (EbSh-5, EbSh-13, EbSh-23, EbSh-36, WB02, WB08). Absolute elevation accuracy was tested by comparing DSM height at the water’s edge against predicted tidal heights at the time of image capture. SfM survey accuracy typically degrades over larger areas [21]. Therefore, tidal stage reference points were added to boundary areas located more than 100m away from GCP targets to improve vertical accuracy. Other factors could alter water levels at the time of data collection such as wind and sea state; therefore, we estimate that absolute elevation accuracy is < 20 cm for sites located more than 100 m from our control locations (KB14-05, EbSh-58, EbSh-77).

## 3.0 Results

Of the nine clam gardens sampled and dated in Kanish and Waiatt Bays, seven sites represent Form 1 clam gardens on soft sediment beaches (EbSh-5, EbSh-13, EbSh-58, EbSh-36, KB14-05, EbSh-77, EbSh-23), while two represent Form 3 gardens on previously steep and eroding shorelines (WB02, WB08). Due to unclear stratigraphy and high water tables we were not able to collect suitable dating samples from the Form 2 gardens. Two Form 1 study sites contained multiple walls and terraces (EbSh-5, EbSh-58 Fig 3D and 3E). In total, we analyzed sixty-one radiocarbon samples from the nine sites (S1 Table, S2 Table). We divided these in two groups: samples which are unsuitable for determining when clam gardens were built and/or used, and those which are suitable. All samples have allowed us to establish sampling guidelines for dating clam gardens (S1 File) in areas of falling sea levels. We discuss each group of unsuitable and suitable samples below followed by a discussion of how the relative sea level history and tidal heights of the samples and walls can also be used to ascertain clam garden ages. All radiocarbon dates discussed in the text are the median calibrated (cal BP) dates.

### 3.1 Unsuitable samples

Twenty-six samples from complicated depositional contexts could not be tied definitively to clam garden construction or use and thus were removed from our age assessments of the gardens (S1 Table). These samples do, however, help us understand the best contexts for dating clam gardens (S1 File). The samples fall into four different contexts. The first set is composed of very young samples from within and below the walls that were extracted too close to the wall exterior to offer insight into wall construction or use (sample #141822, 132186, 145719, 132185). That is, we realized that modern juvenile shellfish and barnacle spat can easily settle within the air spaces between the outer more exposed boulders. Our inference that this zone is biologically active is reinforced by the fact we found live clams up to 1.25 m horizontally in from the toe (seaward edge) of the wall. As we came to understand clam garden construction, we directed our sample collection to the base of those walls or parts of walls that were several rocks tall (preferably in excess of 30 cm—the maximum burrowing depth of clams), densely packed, and encased in sediment to avoid recent clams that may have burrowed into or under the wall.

A second set of removed samples are those that are in secondary contexts and thus pre-date the wall construction event. For example, we learned that clam samples most useful for dating are those that died in growth position (i.e., paired valves with umbo pointing down or sideways). In two instances, we sampled clams from within the wall that were not in growth position (#132187, 163684). Their ages are older than other clams from secure dating contexts below the same section of wall, leading us to hypothesize that people used the older clams as wall-fill along with the pebbles and cobbles that are common wall building materials (Fig 4). The third set of removed samples comes from shellfish specimens that we later identified as, or found associated with, invasive species and were buried within the terrace as a result of recent sedimentation perhaps associated with 20^th^ century logging (#141819, 141820).

The final group of removed samples are barnacle scars (#159609, 159610, 132182) and clams (#159608, 159611, 159612, 171654, 171655, 171656, 132182, 141813, 145722, 159607, 171657, 171658, 163685, 163686, 171661, 145723) from distinct paleobeach deposits located well below (and dating much earlier than) the clam garden walls and terraces. These clam samples represent early Holocene specimens that were lying on or within the old beach surface for thousands of years before being buried by the clam garden wall or sediments. As our sample size increased, we realized that these older “paleo-shells” could often be identified *a priori* in the field because they were usually part of a thick death assemblage of clams. We surmise that these death assemblages form during periods of relative sea level stasis, when beach sediments and organisms accumulate in the paleo-environmental record. The paleo-shells are a fundamental component of our study reconstructing the shellfish ecology of each beach prior to clam garden construction [9, 22, 23] and have been used to help refine late Pleistocene and early Holocene sea level history [13], but play only a limited role in this discussion on dating clam gardens. We suspect that some of our later Holocene samples (4,000–3,000 year range) discussed below may have also accumulated during a period of relative sea level stasis, just prior to or concomitant with construction of the first clam gardens.

### 3.2 Suitable samples for constraining age of clam garden construction and/or subsequent use

**Fig 6:**
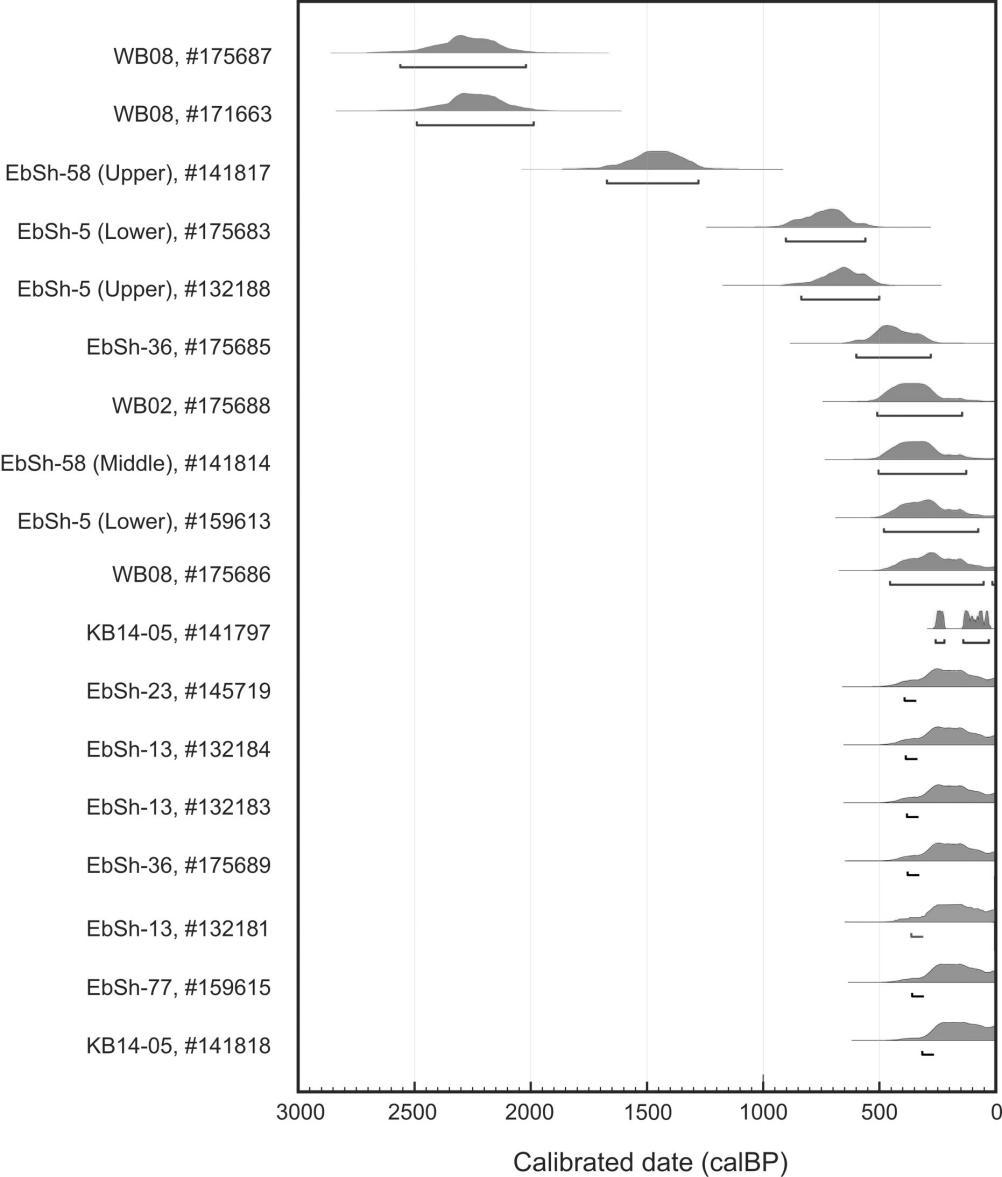
Clam garden use dates: Oldest dates from within wall or terrace, or immediately below terrace sediments at each clam garden site. Radiocarbon calibrations and curve produced in OxCal 4.3 [26], IntCal 13 atmospheric and Marine 13 marine [27].

**Fig 7:**
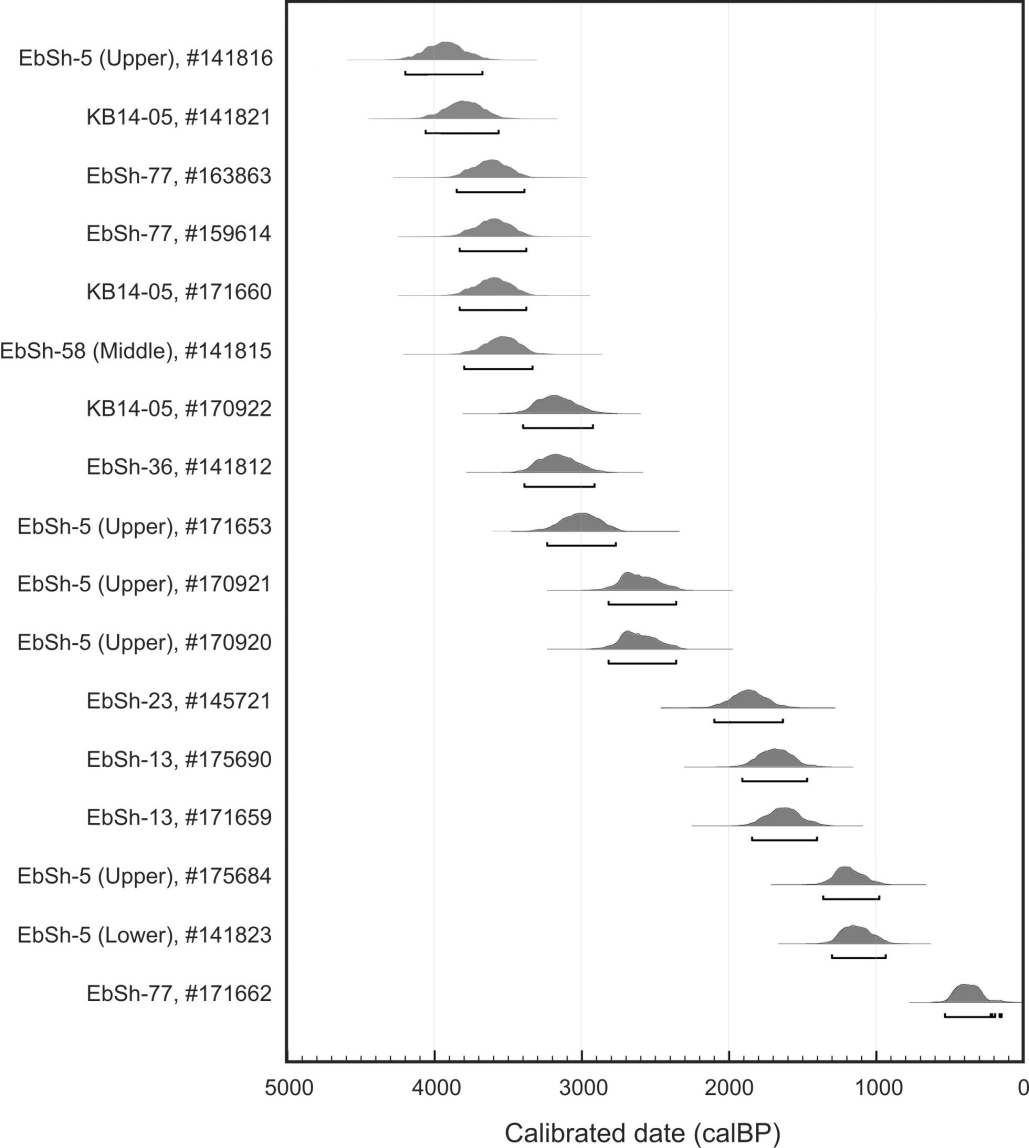
Clam garden below wall dates. Note there are no below wall samples from sites WB08 and WB02 as these are Form 3 clam gardens and wall bases were inaccessible. Radiocarbon calibrations and probability plots produced with OxCal 4.3 [26]. IntCal 13 atmospheric and Marine 13 marine [27].

To date clam garden construction and use, we consider samples from within the walls and the terraces (N = 15), from immediately below the terraces (N = 3), and from immediately below the walls (N = 17) (S2 Table, Figs 6 and 7). Taken together, these samples span a period from ~4000 years ago to AD 1950 (the end of the radiocarbon curve).

Samples recovered from the terraces or within the walls were deposited sometime during the use of the garden. The majority of dates from within the walls and terraces span the last 700 years (N = 13, sample types 2b and 3 in S2 Table and S1 File), though two are from ~2200 BP (#175687 and #171663) reflecting a tendency for older clams to be removed through harvesting. The relatively recent dates collectively reflect continuous use of the clam gardens up until the 20^th^ century, and are consistent with Keekus’ comment noted earlier about harvesting in clam gardens in her lifetime.

Three additional samples that age the use of clam gardens include a barnacle scar (#132181) and a whelk (#141817, 175688) found immediately below the clam garden terrace sediments and in shovel tests well inshore and upslope of the wall (sample type 4 in S2 Table and S1 File). These samples range in age from ~1460 to ~180 cal BP. While these samples are below the terrace sediments, and in the cases of samples 132181 and 141817, on the surface of the former pre-garden beach, we consider these samples to be use dates as it would have taken some unknown amount of time after wall construction for these samples to be covered by sediment. Determining when these samples were deposited relative to initial wall construction requires understanding how terraces were in-filled with sediment post-wall construction. Our current hypothesis is that sedimentation first occurred behind the wall and then progressed upslope; however we do not know the rate or pattern of this progression.

The below wall samples are composed of clams, whelks, and a limpet that were trapped during wall construction when the first boulders were placed at the base of the walls. As such, these samples provide lower constraining ages for clam garden wall construction (sample types 5 and 6 in S2 Table and S1 File; Fig 7). In our study, these samples fall within two time periods. The first are a set of specimens (N = 6) from below the wall that date to within the last 1900 years, with the majority falling between 1700–1100 cal BP (Fig 7). Three of the six specimens from below the wall are non-burrowing organisms and would have lived and died on the beach surface. Because these three are epifaunal we can be confident that they date the old beach surface at or near the time of wall construction.

The second set of below wall samples (N = 11) dating between 2600–3950 cal BP consist of butter clams and one littleneck clam. Taken alone we cannot assume these samples represent the time of wall construction as mollusc shells can survive in normal intertidal conditions for as much as 4000 years [24]. This extended survivorship of datable material creates an “old shell effect” such that it can be difficult to associate temporally the death of an organism with another event. While these samples do offer a constraining age for clam garden construction, additional evidence is needed to support a hypothesis that the walls could have been built during this older period. For that we turn to RSL of the study area.

### 3.3 Using relative sea level data and tidal heights to evaluate clam garden ages

**Fig 8:**
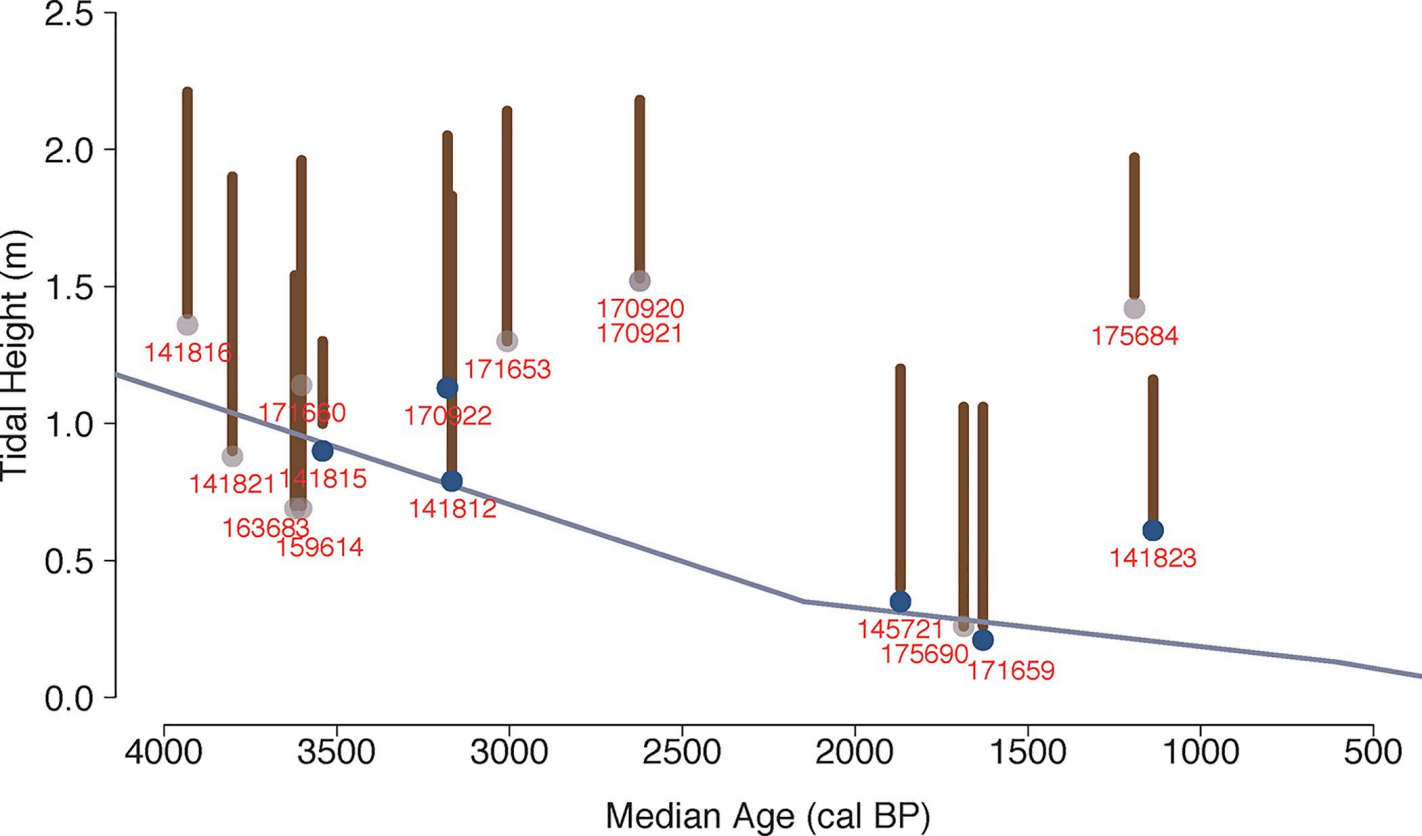
Position of mid-late Holocene (4000–500 cal BP) median clam dates from below the walls (closed circles) and next to walls (open circles), relative to top and bottom of walls (vertical lines) and to sea level (blue trend line; Crowell 2017).

To determine whether the mid-to-late Holocene clam samples from below the walls are associated with clam garden initiation and use, we compare elevation data for the dated samples, for the top and bottom of the walls, and for the relative position of dropping sea level post-4000 years ago [14] (Fig 8). We also consider the elevations of the walls in relation to the maximum ideal tidal zone of littleneck and butter clams (up to 1.3 m and 0.5 m above chart datum (LLWLT) respectively [20, 25]).

To be accepted as an accurate clam garden date, a below wall sample must meet three criteria: 1) given that walls were built at the lowest low tide line, the elevation of the wall bases and that of the dated clam sample should be at or near sea level at the time of construction; 2) the wall and terrace height should be within the most productive butter clam and little neck zone for the sea level at the time, and, given falling sea level, if the garden dates to an earlier sea level, the wall height should be at a higher elevation and in some cases out of the productive clam zone today; and finally, 3) there should be no younger clam date from below the same wall, which would indicate that the older date reflects the “old shell effect”.

Based on these criteria, we are confident that six shell dates representing six different gardens (Fig 8) correspond with the time of clam garden wall construction. Particularly striking is that the bases of the oldest walls are higher than the tops of some of the more recent walls. Collectively, these indicate that clam gardens were initiated at least 3500 years ago, and that new clam gardens were built at various times over the last 2300 years.

We have selected these criteria to be deliberately conservative in our assessment of the age of clam gardens in our study area. As a result, we suspect that some of the discounted dates may reflect clam garden building events. For instance, our first criterion about wall height does not take into account the possibility of settling along all or parts of the wall through auto-compaction, or tectonic activity [14]. In addition, criterion 3 assumes that all wall building within a garden was contemporaneous. However, the array of different dates from the upper walls at site EbSh-5 (Fig 5) suggests a series of building events at different times in the past, and that an earlier initial construction for the upper wall around 3000–4000 cannot be ruled out (Fig 5). Furthermore, the site’s unique geomorphological context (a tombolo), likely necessitated complex engineering considerations that are not encompassed by our three general criteria, perhaps accounting for the unusual tidal heights of samples at EbSh-5 (Fig 8).

Our below wall dates fall into two time periods: pre- and post- 2300 cal BP (N = 2 and 3, respectively) with a gap of ~400 years between the two sets of below wall dates (Fig 8). We hypothesize that this gap is associated with a tectonic event some time between 2600–2200 years ago that caused the land to subside. This hypothesis is supported by independent dating of terrestrial middens which demonstrate that samples on soft sediments that pre-date 2200 cal BP are now below current sea level [14].

Samples in grey are those that do not meet all three of our criteria for sample verification. Note that none of the samples from the upper wall at EbSh-5 (samples 141816, 171653, 170920, 170921, 175684) meet our criteria for sea level and tidal height data. However, we suspect this is a factor of local geomorphology and a complex history of wall building. Terrace dates further from the wall are not shown as these do not tie closely to the time of wall initiation, but rather to later use. We use median dates for ease of presentation, yet note that plotting the calibrated date range would not change our results.

## 4.0 Discussion

Our dating program allows us to generalize about the best contexts to age clam gardens under a variety of geomorphological and ecological settings (see S1 File for sampling guidelines) and demonstrates that meaningful age determinations can be established for clam garden terraces, despite being primarily inorganic constructions. Clam gardens are complex living landscapes subject to continuous abiotic, biotic, and cultural processes. As such, there is value in exploring multiple sampling contexts to understand the chronology of clam garden construction and use, and in considering the role of local sea level histories in formulating those chronologies. Our proposed methodology works in places where sea level is dropping, such as the Quadra Island study area, but elsewhere the details of sampling may need refinement to suit the local sea-level history.

As a result of our suite of dates from diverse contexts, we can make some interpretations about the age of clam gardens in Kanish and Waiatt Bays. Prior to ~2500 years ago, the correspondence of sea level with wall height and below wall clams suggests that clam gardens in our study area are at least ~3500 years old. These ages are in line with the ages of other archaeological marine management features on the Northwest Coast [28, 29] and extends the ages for clam gardens reported previously [8, 12].

In a previous paper, our team reported on results using optically stimulated luminescence (OSL) dating to date clam garden features [12]. Our OSL dates on sediments from the terrace and within the wall of site KB14-05 and EbSh-58 indicated that clam gardens were built and used ~1200–1900 years ago,–nearly two thousand years younger than the radiocarbon and sea level estimates from the same deposits reported here (samples 170922, 141815 S2 Table). Our on-going research into OSL dating protocols in this region suggests that similar temporal discrepancies between C-14 versus OSL exist elsewhere on Quadra Island, and may reflect the need to re-evaluate OSL protocols for our study area. [30]. This suggests that the methodological approach outlined in the previous publication for sampling sediments in mariculture and agricultural terrace features is most applicable in areas where the comparability of OSL and radiocarbon results have been well established.

The more recent set of below wall and terrace dates indicate that clam gardens were (re)constructed continuously from ~2200 cal BP to 340 cal BP. The mid-wall and terrace dates, combined with Keekus’ statements about digging clams in Waiatt Bay, extends the use of clam gardens in our study area into the historic period. Today, Indigenous clam diggers, some of who have knowledge of traditional clam garden maintenance and use, harvest from the clam garden beaches of northern Quadra. One of the authors of this paper, in fact, is an active clam digger–and thus is carrying on the tradition into modern contexts.

People’s creation of clam gardens, and an understanding of the development of clam gardening more generally, is intertwined with a detailed understanding of local sea levels and local geomorphology. We suspect that our current understanding of wall construction is an artefact of us targeting both the garden walls that are highest in the intertidal (and thus presumably the oldest) as well as the more recent clam gardens associated with current sea levels. The many clam gardens in our study area that are at or near current sea level are likely the end product of years of dismantling and moving walls toward the sea as sea level dropped through time; this process results in older walls being harder to identify. In some gardens, recent refurbishment is reflected in small, unfinished walls at the current low-low tides. The construction of these small walls, however, seems to have halted with dramatic population decline associated with the introduction of European diseases into the region.

Documenting the specific timing of clam garden initiation and use is fundamental to understanding the social-ecological history of Kanish and Waiatt Bays. In these bays, people seemingly augmented or created clam habitat in every bit of available foreshore, and ultimately increased the area of clam habitat by up to ~50% [9]. People took a simple construction method (rolling rocks to the lower intertidal) and adapted it to a variety of geomorphological settings resulting in the three forms we have identified here. It is rare in the archaeological record to have such tangible evidence of long-term management systems and to be able to trace their development through time [31]. However, while we have identified clam gardens extending back 3,500 years, we must be aware that older walled structures associated with higher sea levels may be present upslope in today’s dense rainforest. Similarly, building rock-walled beaches was only one component of ancient shellfish mariculture. Our data are largely silent with respect to the majority of management practices (e.g., tilling, predator removal, harvesting proscriptions) that likely occurred within both clam gardens and non-walled beaches. Such practices, and the associated social and cultural belief systems in which they were embedded, almost certainly have deeper historical roots than we can measure by the presence or absence of rock-faced beach terraces. Archaeologists and paleoecologists need to be cognizant of the biases in the paleo-record towards these more tangible aspects of what were once highly complex, age-old, management systems.

Our detailed dating of clam gardens provides the foundation for exploring how mariculture in Kanish and Waiatt Bays developed in relation to other social and economic shifts. One expectation, based on classic models of resource intensification [32], is that people put increased effort into improving clam production via clam gardens when spurred on by some social and/or ecological factor (e.g., increasing human population, increasingly reified systems of ownership and control, natural declines in clam populations). We might further predict that the inhabitants would put effort first into the already productive clam beaches and only turn to the less productive locations when forced to do so. Contrary to this prediction, the oldest dated clam gardens in our sample are on small, steep beaches, away from primary settlements. It is possible, however, that the clam garden walls that were originally built on the more naturally productive and easily enhanced beaches were deconstructed and remodelled as sea level dropped. This would especially be the case on beaches associated with large settlements (e.g., EbSh-13), where unused walls in the upper intertidal would impede daily activities. That different portions of long walls likely date to different times (e.g., at EbSh-5), indicates that garden walls were likely refurbished as social and ecological contexts shifted. Our ongoing analysis will examine the apparent iterative relationship between large settlements and the initiation of clam gardens.

The lessons learned from our dating program are relevant to the dating of other intertidal features elsewhere in the world. In particular, many of the same considerations applied here would be helpful for dating rock walled fish traps where no wooden elements have preserved. In such cases, radiocarbon samples of invertebrates trapped underneath the rock walls will provide a constraining date for the building of that wall section. Surface dwelling invertebrates with less durable shells or skeletal elements will be less affected by the old shell effect. Moreover, in areas of falling sea level where intertidal features may be stranded in the intertidal or on land, or in areas of rising sea levels where walls are submerged, relict walls may best be dated using a well defined sea level curve (e.g., [13, 33, 34].

When investigating any traditional marine management system, researchers should be cognizant that Indigenous traditional ecological knowledge systems often incorporate a long-term view of resource management [1, 35]. In the case of clam gardens, this is reflected in local knowledge [6] and our OSL vertical cores [12], which indicate that garden walls were built up slowly over time and that the terraces filled in gradually, over generations of use. Although some ecological benefit was accrued by those first wall builders, the full benefit of clam gardens (i.e., increasing clam productively up to fourfold) was not experienced until many generations later. This kind of future-focussed management practice, that ensures food security for generations to come, stands in sharp contrast to the short-sighted way that many of our natural resources are managed in today’s industrial age.

Taken together, our investigations provide glimpses into the social context in which clam gardens were constructed and maintained, and indeed in which clams were cultivated and managed. In some cases, as indicated by the carefully placed non-intertidal rocks at the base of the walls, the initial garden wall foundation was constructed in single well-coordinated construction event (e.g., Form 1 clam garden EbSh-5; Fig 4). In other cases, less well-constructed wall foundations (i.e., of loose rubble fill) make it difficult to distinguish those initial construction events from the on-going rock-rolling involved in clam garden maintenance [6]. We surmise, however, that such planned initiation events were commonplace, especially in Form 2 and 3 gardens where rock placement would have had more rapid returns (i.e., by creating clam habitat where there were was none prior). In imagining those initial construction events, and the on-going build up of the rock walls through the deliberate rolling of rocks, we can imagine how labour was organized, the kinds of ecological and engineering knowledge held by at least some community members, and how knowledge was passed down and shared. These social histories, played out over and over again through the generations, are imprinted on and embedded in the crevices of the rock walls that we see today.

## Supporting information

S1 Table Unsuitable samples for clam garden age assessments. (DOCX)

S2 Table Radiocarbon determinations used to constrain ages or date Quadra Island clam garden sites. Bolded dates are oldest accepted date for each garden. Refer to S1 File for key to sample type.
(DOCX)

S1 File Sampling Guidelines for Dating Clam Gardens. Includes: **Idealized radiocarbon sample types from a clam garden (Figure A).** (a) Form 1 clam garden b) Form 2 clam garden c) Form 3 clam garden. Potential sampling locations may shift depending on local sea level histories. Refer to Table A for description of sample location numbers, their context, and potential interpretations. **Types of radiocarbon sampling contexts for clam gardens (Table A).**
(DOCX)
