# Supplementary material for: 3500 years of shellfish mariculture on the Northwest Coast of North America
Source: PLoS One. 2019 Feb 27;14(2):e0211194. doi: 10.1371/journal.pone.0211194 (PMC6392220; doi:10.1371/journal.pone.0211194)
Supplement: S1 Table — (DOCX) [file pone.0211194.s001.docx]

S1 Table. Samples not used in clam garden age assessments

| **Site no.** | **UCI**  **lab no.** | **Test no.** | **Surface Elev. of test** | **Sample DBS** | **Tidal ht of sample above LLWLT** | **Material** | **Conventional C14 age** | **Cal age BP**  **(2 sigma)^3^** | **Median age** | **Reason not used** |
| --- | --- | --- | --- | --- | --- | --- | --- | --- | --- | --- |
| EbSh-5 (Upper) | 132187^1^ | Trench 2 | 1.97 | 0.50 | 1.5 | butter clam | 2545 +/- 25 | 1588 - 2057 | 1825 | Clam not in growth position = wall fill |
| EbSh-5 (Upper) | 171654^2^ | Trench GT08 | 2.14 | 1.05 | 1.1 | butter clam | 10405 +/-25 | 10632 -11007 | 10796 |  |
| EbSh-5 (Upper) | 171655^2^ | Trench GT08 | 2.14 | 1.30 | 0.8 | butter clam | 10715 +/-25 | 11028 - 11280 | 11164 |  |
| EbSh-5 (Upper) | 171656^2^ | Trench GT08 | 2.14 | 1.40 | 0.7 | butter clam | 10665 +/-25 | 10955 - 11236 | 11121 |  |
| EbSh-77 | 163684 | Trench 1 | 1.54 | 0.70 | 0.8 | butter clam | 4460+/-15 | 3932 - 4441 | 4206 | Clam not in growth position = wall fill |
| EbSh-77 | 145723 | Trench 1 | 1.78 | 0.90 | 0.7 | butter clam | 6110+/-25 | 5975 - 6396 | 6201 |  |
| EbSh-23 | 145720 | Trench 1 | 1.20 | 0.85 | 0.4 | littleneck | 935 +/- 25 | 46 – 435 (0.971) | 248 | Sample not far enough from toe of wall. Live clams noted in sampling area |
| EbSh-13 | 132186^1^ | Trench WTA | 1.24 | 0.92 | 0.3 | barnacle scar | -50 +/-25 | N/A (negative C14 age) |  | Sample not far enough from toe of wall |
| EbSh-13 | 132185^1^ | Trench WTA | 1.24 | 0.92 | 0.3 | barnacle scar | 980 +/- 25 | 73 - 483 | 303 | Sample not far enough from toe of wall |
| EbSh-13 | 132182^1^ | Terrace Trench | 1.56 | 0.53 | 1.0 | barnacle scar | 4075 +/-25 | 3445 - 3928 | 3691 |  |
| KB14-05 | 141822 | Trench 1 | 1.90 | 0.72 | 1.2 | Jingle shell | -5+/-20 | NA |  | Invalid age for curve. Sample not far enough from toe of wall |
| KB14-05 | 141819 | ST1  (terrace) | 1.94 | 0.37 | 1.6 | mya | -80+/-20 | NA |  | Invalid age for curve. Invasive species. in upper deposit likely associated with historic logging |
| KB14-05 | 141820 | ST1  (terrace) | 1.94 | 0.40 | 1.5 | littleneck | 385+/-20 | NA |  | Invalid age for curve. In upper deposits associated with invasive species, likely influenced by historic logging |
| KB14-05 | 171661 | Trench 1 | 1.90 | 0.81 | 1.1 | butter clam | 7975+/-20 | 7938 - 8314 | 8116 |  |
| KB14-05 | 163685^2^ | Trench GT07 | 1.96 | 1.00 | 1.0 | butter clam | 9380+/-25 | 9531 - 10089 | 9779 |  |
| KB14-05 | 163686 | Trench GT07 | 1.96 | 1.00 | 1.0 | oyster or jingle shell | 9470+/-20 | 9622 -10170 | 9915 |  |
| EbSh-36 | 141813^2^ | Trench 1 | 1.83 | 1.08 | 0.8 | butter clam | 9960+/-30 | 10247 – 10746 | 10521 |  |
| EbSh-36 | 145722^2^ | Trench 1 | 1.83 | 1.05 | 0.8 | butter clam | 9605+/-35 | 9771 – 10348 | 10088 |  |
| EbSh-36 | 159607^2^ | ST2  (terrace) | 1.85 | 0.70 | 1.2 | butter clam | 10295+/-25 | 10501 – 10827 | 10649 |  |
| EbSh-36 | 171657^2^ | ST2 (terrace) | 1.85 | 0.26-0.31 | 1.6 | butter clam | 9890+/-20 | 10218 – 10651 | 10435 |  |
| EbSh-36 | 171658^2^ | ST2 (terrace) | 1.85 | 0.44 | 1.4 | butter clam | 10085+/-20 | 10239 – 10536 | 10393 |  |
| EbSh-36 | 159609^2^ | ST2  (terrace) | 1.85 | 0.89 | 1.0 | barnacle scar | 10555+/-25 | 10789 - 11150 | 10996 | From paleobeach deposit well below clam garden terrace. Indicates conditions changed suddenly to enable preservation (possibly rapid sedimentation, submergence or tectonic event) |
| EbSh-36 | 159610^2^ | ST2  (terrace) | 1.85 | 0.89 | 1.0 | barnacle scar | 10570+/-25 | 10808 - 11164 | 11016 | From paleobeach deposit well below clam garden terrace. Indicates conditions changed suddenly to enable preservation (possibly rapid sedimentation, submergence or tectonic event) |
| EbSh-36 | 159608^2^ | ST2  (terrace) | 1.85 | 0.95-1.05 | 0.9 | butter clam | 10485+/-20 | 10719 - 11084 | 10903 | From paleobeach. |
| EbSh-36 | 159611^2^ | ST5  (seaward of wall) | 0.83 | 0.45-0.50 | 0.4 | littleneck | 10250+/-25 | 10444 - 10752 | 10600 | Test in paloebeach seaward of clam garden wall |
| EbSh-36 | 159612^2^ | ST5  (seaward of wall) | 0.83 | 0.45-0.50 | 0.4 | butter clam | 10065+/-25 | 10224 - 10517 | 10368 | Test in paleobeach seaward of clam garden wall |

1. Lepofsky et al. 2015
2. Toniello et al. 2015, 2016; Fedje et al. 2018
3. Calibration using Calib 7.1 (Reimer et al. 2013) with a marine Delta R of 320+90 for post-10,000 ^14^C BP samples and 550+50 for pre-10,000 ^14^C BP samples (Hutchinson et al. 2004).
