# Supplementary material for: 3500 years of shellfish mariculture on the Northwest Coast of North America
Source: PLoS One. 2019 Feb 27;14(2):e0211194. doi: 10.1371/journal.pone.0211194 (PMC6392220; doi:10.1371/journal.pone.0211194)
Supplement: S2 Table — Bolded dates are oldest accepted date for each garden. Refer to S1 File for key to sample type. (DOCX) [file pone.0211194.s002.docx]

S2 Table. Radiocarbon determinations used to constrain ages or date Quadra Island clam garden sites. Bolded dates are oldest accepted date for each garden. Refer to S3 for key to sample type.

| **Site No.** | **Garden Form** | **UCI**  **Lab No.** | **Sample Type** | **Test No.** | **Surface elev of test (wall max)** | **Base of wall elev (wall min)** | **Sample DBS** | **Sample Tidal Ht Above LLWLT^1^** | **Material^2^** | **Conventional C14 Age** | **Cal Age BP**  **(2 sigma)^3^** | **Median age** |
| --- | --- | --- | --- | --- | --- | --- | --- | --- | --- | --- | --- | --- |
| EbSh-5  (Upper) | 1 | 132188**^4^** | 2b | Trench 2 | 1.97 | 1.47 | 0.44 | 1.5 | butter clam | 1410 +/- 25 | 502 - 836 | 656 |
| **EbSh-5 (Upper)** | **1** | **175684** | **6** | **Trench 2** | **1.97** | **1.47** | **0.55** | **1.4** | **butter clam** | **1960 +/-15** | **980 - 1362** | **1192** |
| EbSh-5 (Upper) | 1 | 170920 | 6 | Trench CN | 2.18 | 1.53 | 0.66 | 1.5 | butter clam | 3200 +/-15 | 2360 - 2820 | 2623 |
| EbSh-5 (Upper) | 1 | 170921 | 6 | Trench CN | 2.18 | 1.53 | 0.66 | 1.5 | butter clam | 3200 +/-15 | 2360 - 2820 | 2623 |
| EbSh-5 (Upper) | 1 | 171653 | 6 | Trench GT08 | 2.14 | 1.3 | 0.82 | 1.3 | butter clam | 3520 +/-15 | 2770 - 3238 | 3007 |
| EbSh-5 (Upper) | 1 | 141816 | 6 | Trench 2014 | 2.21 | 1.4 | 0.8-0.9 | 1.3-1.4 | butter clam | 4260 +/-20 | 3677 - 4198 | 3932 |
| EbSh-5 (Lower) | 1 | 175683 | 2b | Trench 1 | 1.16 | 0.61 | 0.5 | 0.7 | butter clam | 1490+/-15 | 562 - 903 | 729 |
| EbSh-5 (Lower) | 1 | 159613 | 3 | ST12 | 1.25 | - | 0.42 | 0.8 | littleneck | 980+/-15 | 78 - 481 | 304 |
| **EbSh-5 (Lower)** | **1** | **141823** | **6** | **Trench 1** | **1.16** | **0.61** | **0.5-0.55** | **0.61 – 0.66** | **butter clam** | **1905+/-20** | **938 – 1300** | **1138** |
| EbSh-13 | 1 | 132181**^4^** | 4 | Terrace Trench | 1.56 | - | 0.32 | 1.2 | barnacle scar | 870 +/-25 | 0 - 361 | 177 |
| EbSh-13 | 1 | 132183**^4^** | 3 | Terrace Trench | 1.56 | - | 0.24 | 1.3 | butter clam | 890 +/-25 | 0 - 382 | 195 |
| EbSh-13 | 1 | 132184**^4^** | 3 | Terrace  Trench | 1.56 | - | 0.24 | 1.3 | butter clam | 895 +/-25 | 0 - 387 | 200 |
| **EbSh-13** | **1** | **171659** | **5** | **Trench GT09** | **1.06** | **0.26** | **0.85** | **0.2** | **whelk** | **2375 +/- 15** | **1404 - 1844** | **1630** |
| EbSh-13 | 1 | 175690 | 6 | Trench GT09 | 1.06 | 0.26 | 0.8 | 0.3 | macoma | 2425 +/- 15 | 1472 - 1910 | 1686 |
| **EbSh-58**  **(Upper)** | **1** | **141817** | **4** | **STC** | **2.20** | **-** | **0.4** | **1.8** | **whelk** | **2225+/-20** | **1279 - 1673** | **1458** |
| EbSh-58  (Middle) | 1 | 141814 | 3 | STB | 1.44 | - | 0.36 | 1.1 | butter clam | 1020+/-20 | 130 - 503 | 344 |
| **EbSh-58**  **(Middle)** | **1** | **141815** | **6** | **Trench 1** | **1.3** | **1.0** | **0.4** | **0.9** | **butter clam** | **3955+/-25** | **3336 - 3799** | **3541** |
| EbSh-36 | 1 | 175689 | 3 | ST LN1 | 1.92 | - | 0.55-0.45 | 1.4.-1.5 | littleneck | 890+/-15 | 0 – 379 | 194 |
| EbSh-36 | 1 | 175685 | 3 | ST NS2 | 1.96 | - | 0.38 | 1.6 | littleneck | 1140+/-15 | 280 - 599 | 442 |
| **EbSh-36** | **1** | **141812** | **6** | **Trench 1** | **1.83** | **0.82** | **1.04** | **0.8** | **butter clam** | **3645+/-20** | **2915 – 3390** | **3167** |
| KB14-05 | 1 | 141797 | 3 | ST SW | 1.95 | - | 0.47 | 1.5 | wood | 100+/-15 | 31 – 48 (0.129)  53 –138 (0.583)  223 - 257 (0.288) | 114 |
| KB14-05 | 1 | 141818 | 3 | ST SW | 1.95 | - | 0.46 | 1.5 | littleneck | 845+/-15 | 0 - 318 | 157 |
| KB14-05 | 1 | 141821 | 6 | Trench 1 | 1.90 | 0.90 | 1.02 | 0.9 | butter clam | 4165+/-20 | 3567 - 4061 | 3802 |
| **KB14-05** | **1** | **170922** | **6** | **Trench CN** | **2.05** | **1.15** | **0.92** | **1.1** | **littleneck** | **3655+/-15** | **2928 - 3399** | **3180** |
| KB14-05 | 1 | 171660 | 6 | Trench GT07 | 1.96 | 1.16 | 0.82 | 1.1 | butter clam | 4005+/-15 | 3378 - 3831 | 3602 |
| EbSh-77 | 1 | 159615 | 3 | ST2 | 1.53 | - | 0.32 | 1.2 | littleneck | 870+/-15 | 0 – 339 (0.986) | 177 |
| **EbSh-77** | **1** | **171662** | **5** | **Trench 1** | **1.54** | **0.7** | **0.80** | **0.7** | **limpet** | **1065+/-15** | **224 – 535 (0.986)** | **380** |
| EbSh-77 | 1 | 159614 | 6 | Trench1 | 1.54 | 0.7 | 0.85 | 0.7 | butter clam | 4005 +/- 15 | 3378 – 3831 | 3602 |
| EbSh-77 | 1 | 163683 | 6 | Trench1 | 1.54 | 0.7 | 0.85 | 0.7 | butter clam | 4020 +/- 15 | 3392 - 3850 | 3621 |
| EbSh-23 | 1 | 145719 | 3 | ST1 | 1.40 | - | 0.27 | 1.3 | butter clam | 900 +/- 25 | 0 - 392 | 205 |
| **EbSh-23** | **1** | **145721** | **5** | **Trench 1** | **1.20** | **0.4** | **0.85** | **0.4** | **whelk** | **2580 +/- 25** | **1631 - 2104** | **1869** |
| **WB08** | **3** | **175687** | **3** | **ST NS6** | **0.70** | **-** | **0.38** | **0.3** | **macoma** | **2920 +/- 15** | **2025 – 2563** | **2277** |
| WB08 | 3 | 171663 | 3 | ST1 | 0.96 | - | 0.70 | 0.3 | butter clam | 2895 +/- 15 | 1988 – 2492 | 2243 |
| WB08 | 3 | 175686 | 3 | ST NS6 | 0.70 | - | 0.35 | 0.4 | littleneck | 950 +/- 15 | 53 – 455 (0.988) | 268 |
| **WB02** | **3** | **175688** | **4** | **ST NS3** | **1.11** | **-** | **0.40** | **0.7** | **whelk** | **1040 +/- 15** | **172 - 510 (0.983)** | **361** |

1. Modern tidal range between chart datum and HHWLT is 4.64 m at Waiatt Bay, 4.82 m at Kanish Bay. The average tidal range for Quadra Island is 4.83 (Data provided by Government of Canada, Canadian Hydrographic Service or <http://www.tides.gc.ca)>;
2. All dated clams were found in growth position.
3. Calibration using Calib 7.1 (Reimer et al. 2013) with a marine Delta R of 320+90 for post-10,000 ^14^C BP samples and 550+50 for pre-10,000 ^14^C BP samples (Hutchinson et al. 2004).
4. Lepofsky et al. 2015
