# Supplementary material for: 3500 years of shellfish mariculture on the Northwest Coast of North America
Source: PLoS One. 2019 Feb 27;14(2):e0211194. doi: 10.1371/journal.pone.0211194 (PMC6392220; doi:10.1371/journal.pone.0211194)
Supplement: S1 File — Includes: Idealized radiocarbon sample types from a clam garden (Figure A). (a) Form 1 clam garden b) Form 2 clam garden c) Form 3 clam garden. Potential sampling locations may shift depending on local sea level histories. Refer to Table A for description of sample location numbers, their context, and potential interpretations. Types of radiocarbon sampling contexts for clam gardens (Table A). (DOCX) [file pone.0211194.s003.docx]

**S1 File: Sampling Guidelines for Dating Clam Gardens**

Our testing and dating of nine clam garden sites on Quadra Island allow us to identify sample types that are useful for estimating the time of clam garden construction and use (Table A, Figure A). Below we describe useful radiocarbon sampling types and contexts and consider how tightly we can constrain the time of wall construction or maintenance using radiocarbon dating. Note that this guide has been developed in an area of falling sea level throughout the Holocene and may need modification to suit clam garden sites in areas with transgressing or variable sea level histories.

**S1.1 Dating clam garden initiation and subsequent use**

*Type 1: Barnacle scar at base of wall*

Despite not finding any in our study, the most desirable sample for dating the initiation of a clam garden wall is a radiocarbon dated barnacle scar retrieved from a rock at the base of the garden wall (Table A, Figure A). During clam garden construction, clam diggers would have occasionally placed boulders with live barnacles or barnacle scars in the wall. If the boulders were placed upside down and in sediment that encased them anaerobically, the barnacle scars may preserve. Barnacle scars are ideal targets for radiocarbon dating as they are unlikely to survive on the rock for much longer than a year if exposed to normal intertidal beach conditions [8, 24]. Ideal scars for dating are those found on the underside of a rock and located the base and far from the toe of the wall, where more recent barnacles could not have settled.

In regions where the walls are close to the present day low tide line, or partially submerged, we have had greater success finding barnacle scars [8]. In Quadra Island, however, there are several reasons why the ideal conditions for barnacle deposition and preservation are not often met. First, our wall excavations show that some foundational rocks originated from the upper beach, beyond the barnacle zone (e.g., Fig 4). Second, we found that barnacle scars tend to preserve on mafic, fine-grained rocks. Coarser rocks, such as the granite common in our study area, do not seem to be a suitable substrate for long-lasting barnacle adhesion. Third, since sea level has been falling through the Holocene in Quadra Island [13, 14], the lower portions of the walls are exposed and thus leaving barnacles susceptible to mechanical and biological degradation. Finally, it may be that people have moved the walls from higher positions in the intertidal as sea level fell, further increasing the potential for degradation.

*Types 2a and 2b: Barnacle scar and clam samples from within wall*

As clam gardens are continually maintained, within wall dates can provide temporal information on rebuilding events. Samples collected from higher in the wall and along its outer edges will tend to be younger than those further down and more deeply buried within the wall.

*Type 3: Clam samples from within terrace*

These samples are naturally biased towards more recent time periods because taphonomic processes within the terrace break down shell, and people remove clams as they reach a harvestable size. In our study area, we find death assemblages in terraces that seem to reflect a major disruption or end to the clam garden life cycle. We attribute these disruptions to major environmental events (e.g., falling sea level, inundation of terrigenous sediments) or to the decline in harvesting by Indigenous populations as a result of devastating European-introduced disease epidemics. In some cases, older, unharvested clams can remain near the base of the terrace sediments, particularly in Form 3 gardens. In these cases, clams can become trapped in crevices between boulders and thus presumably would offer protection from people’s digging sticks.

*Type 4: Epifaunal samples from base of terrace in Form 3 garden or top of previous beach surface in Form 1 and 2 gardens*

Type 4 includes whelks and other non-burrowing invertebrates (barnacles, limpets) found on the surface of the previous beach deposits below clam terrace sediments in Form 1 gardens; on the bedrock platform of Form 2 gardens; or at the base of the terrace sediments and among the levelled boulders that form the base of Form 3 gardens. While we do not know how long it took before terrace sediments began accumulating behind clam garden walls and covered these samples, we suspect this process began within a few decades of building the wall foundation, particularly immediately behind the wall. When present in excavation units close to the wall, these samples are among the best sample types for aging early use, and potentially initial use, of a clam garden. As one moves inshore, upslope and away from the wall, the association with initial use declines.

**S1.2 Constraining age of clam garden initiation**

*Types 5 and 6: Samples from below wall*

Type 5 samples are at the interface with the pre-garden beach surface and the base of the wall, and apply to Form 1 and 2 gardens only. Barnacle scars, whelks, and other non-burrowing invertebrates (Type 5) are better chronological markers than molluscs because they denote an old beach surface and, in the case of barnacle scars, are not subject to the large time-averaging problems of molluscs. When preserved immediately below a wall, these non-burrowing invertebrates provide a fairly precise indicator of when the specimen was capped with rocks from the clam garden wall.

Clams found below the wall (Type 6) in pre-existing clam beaches (Form 1 gardens) can yield ages from immediately before construction to those far older than the wall construction. We anticipate the “old shell problem” being especially pronounced in regions that have experienced gradually falling Holocene sea levels. Sampling a number of specimens from immediately below the wall, preferably from multiple trenches, and using the most recent date to constrain the age of garden construction can mitigate this problem. The “old shell problem” should be less of an issue where sea levels have been rising throughout the Holocene as clams colonize new habitat as they progress upslope.





**Figure A Idealized radiocarbon sample types from a clam garden.** (a) Form 1 clam garden b) Form 2 clam garden c) Form 3 clam garden. Potential sampling locations may shift depending on local sea level histories. Refer to Table A for description of sample location numbers, their context, and potential interpretations.

**Table A Types of radiocarbon sampling contexts for clam gardens.**

| **Sample type** | **Specimen dated** | **Sampling Context** | **Target event** | **Comments** |
| --- | --- | --- | --- | --- |
| 1 | Barnacle scar | On rock at base of wall, ideally on the underside of rock preserved in anaerobic sediment. | Initial wall construction, when rock moved from beach to base of wall | Requires excavation trench in wall of Form 1 and 2 clam gardens. Requires 2-3 days of field time for crew of 2-4 people to excavate trench. Preservation is best on fine-grained mafic rocks. |
| 2a | Barnacle scar | On rock within wall | Use of clam garden | Requires excavation trench in wall. 2-3 days of field time for a crew of 2- 4 people. Sample closest to the base of wall will provide oldest age, since the wall height increased over time as people added rocks to the wall. |
| 2b | Clam | Trapped between rocks within wall. Samples must have paired valves and be in growth position to ensure not added to wall as fill. | Use of clam garden |  |
| 3 | Clam | From within the clam garden terrace, above the interface with the prior beach surface. | Use of clam garden | Requires shovel test in terrace. Biased towards recent use of clam garden as ongoing harvesting removes older shells. A death assemblage of clams outside the present day tidal height for clams reflects some disturbance (e.g., falling sea level, inundation with terrigenous sediments, removal of Indigenous care takers who were moved to reserves, sent to residential schools, etc.). |
| 4 | Non- burrowing invertebrate (whelk, limpet, barnacle scar) | Immediately below the clam garden terrace sediments lying on the surface of the previous beach surface. In case of Form 3 collect from base of terrace on part of landform that was newly created during levelling event. | Dates initiation and use of the clam garden; when the terrace sediments accumulated in that part of the garden. | Requires shovel test in terrace. Parts of the terrace closer to the wall may have been covered by sediment sooner than areas upslope and closer to shore. Rate of sedimen-tation will depend on wall height, slope of original beach surface, local geo-morphology. In case of Form 3 clam gardens, take sample from as close to the wall as possible to increase confidence that the terrace did not exist prior to levelling. |
| 5 | Barnacle scar or non-burrowing invertebrate (whelk, limpet, etc) | On surface of old beach, below wall | Pre-garden beach surface just prior to wall construction. Constraining date for clam garden construction. | Requires excavation trench in wall. |
| 6 (C14) | Clam | Below wall | Pre-garden beach surface prior to wall construction. Constraining date for clam garden construction. | Requires excavation trench in wall. As clam shells can preserve on or in beach deposits for thousands of years avoid death assemblages of clams if possible. Shallower burying clams (littlenecks or macomas) preferable to butter or horseclams. |
